# Learning agility, self-efficacy, and resilience as pathways to mental health in higher education: insights from a mixed-methods study

**DOI:** 10.3389/fpsyg.2025.1528066

**Published:** 2025-07-02

**Authors:** He Huang, Heung Kou

**Affiliations:** Department of Education, Graduate School, Sehan University, Yeongam-gun, Republic of Korea

**Keywords:** learning agility, academic self-efficacy, academic buoyancy, psychological well-being, resilience, mixed-methods study, higher education, structural equation modeling

## Abstract

**Introduction:**

This study examines how learning agility, academic self-efficacy, academic buoyancy, and psychological well-being interrelate to influence mental health and factors theoretically linked to academic success in undergraduate students.

**Methods:**

Using an explanatory sequential mixed-methods design, quantitative data were gathered from 804 undergraduates using validated scales for each construct. Structural Equation Modeling (SEM) tested hypothesized relationships, and multi-group analysis explored gender differences in the model. In a qualitative phase, semi-structured interviews with 30 participants provided deeper insights into the quantitative findings, with thematic analysis of adaptive learning, resilience, and well-being.

**Results:**

SEM findings showed that learning agility and academic self-efficacy positively predicted academic buoyancy and psychological well-being, with academic buoyancy partially mediating these relationships. Gender differences were non-significant, supporting model generalizability across genders. Qualitative analysis emphasized adaptive learning strategies, resilience in overcoming academic stressors, and psychological well-being as a process, with social support identified as essential in fostering resilience.

**Discussion:**

The findings underscore the importance of learning agility, self-efficacy, and academic buoyancy in supporting students’ academic resilience and mental health. By enhancing these factors within academic settings, institutions can promote student well-being and engagement, reinforcing the link between psychological well-being and academic achievement.

## 1 Introduction

Research increasingly highlights factors vital for student academic success and well-being amid the challenges of higher education (Kahu and Nelson, 2018; Pintrich and De Groot, 1990; Steinmayr et al., 2018). Students face numerous academic demands, such as unfamiliar content, varying workloads, and critical assessments (Lin and Scherz, 2014; Martin and Marsh, 2020; Putwain et al., 2022), making it essential to understand their coping mechanisms to develop effective support. Four key constructs—learning agility, academic self-efficacy, academic buoyancy, and psychological well-being—each shape how students manage stress and succeed (DeRue et al., 2012; Schunk and DiBenedetto, 2014).

Learning agility, the capacity to rapidly learn from experience and apply insights to new situations, helps students adapt to academic difficulties and cultivate resilience (Lombardo and Eichinger, 2000; Murphy, 2021). Students high in learning agility often engage more effectively with new technologies and interdisciplinary material, which can improve academic performance (De Meuse and Harvey, 2022; Kim et al., 2018). Despite its importance, learning agility research has largely centered on leadership, with less focus on its role in student development (De Meuse et al., 2010; Reed, 2012).

Academic self-efficacy, grounded in Bandura’s (1997) social cognitive theory, signifies students’ confidence in their ability to perform academic tasks. Strong self-efficacy correlates with better academic results, motivation, and persistence, as confident students are more apt to use effective learning strategies and navigate challenges (Kristensen et al., 2023; Schunk, 1991; Usher et al., 2019). Although the link between self-efficacy and academic achievement is well-documented, its interplay with other cognitive-emotional factors like learning agility and resilience warrants further exploration.

Academic buoyancy, or students’ ability to handle routine academic setbacks like poor grades or exam stress, is crucial for sustaining persistence and well-being (Kritikou and Giovazolias, 2022; Martin and Marsh, 2008). Unlike general resilience, which involves overcoming major adversities, buoyancy pertains to managing common, daily academic challenges. Research suggests buoyant students are more likely to stay engaged and maintain mental health despite minor setbacks (Collie et al., 2015; Putwain et al., 2022; Thomas and Allen, 2021). However, how buoyancy interacts with adaptive learning processes, such as learning agility, is not well understood. Similarly, psychological well-being, encompassing emotional health, positive functioning, and self-realization through dimensions like autonomy and personal growth (Ryff, 1995; Ryff and Keyes, 1995; Schmitt et al., 2014), is vital for students facing academic pressure. Higher psychological well-being fosters resilience, enhances academic performance, and lowers risks of anxiety and depression (Amholt et al., 2020; Houben et al., 2015). Its interaction with factors like learning agility and buoyancy in supporting academic success, however, requires further clarification.

Although prior studies have explored relationships among these constructs, limited research has investigated how they collectively influence both academic and psychological outcomes within a unified framework. The mediating role of academic buoyancy between learning agility, self-efficacy, and well-being remains particularly underexplored (Martin and Marsh, 2020; DeRue et al., 2012). Additionally, most research has concentrated on Western contexts, leaving a gap in understanding how these constructs function across diverse educational environments. Given that cultural values significantly shape students’ academic experiences, examining these relationships in broader contexts is essential (Novianti et al., 2023).

This mixed-methods study addresses these gaps by investigating the interplay of learning agility, academic self-efficacy, academic buoyancy, and psychological well-being among undergraduate students in China. Our primary aim is to understand pathways to student mental health, and to explore how these psychological constructs might create a foundation conducive to positive academic experiences. We aim to provide a comprehensive, cross-cultural perspective on these relationships, particularly examining academic buoyancy’s mediating role between learning agility, self-efficacy, and psychological well-being. The study seeks to deepen the understanding of how these cognitive, emotional, and behavioral factors collectively contribute to student mental health outcomes and lay groundwork for future investigations into their direct effects on objectively measured academic achievement.

## 2 Theoretical and empirical background

### 2.1 The role of learning agility in academic success

Learning agility, the ability to rapidly learn from experience and apply knowledge to new situations, is vital for students adapting to evolving academic demands (DeRue et al., 2012; Howard, 2017; Lombardo and Eichinger, 2000). Defined as an experience-driven process, it involves continuous learning and proactive application of insights (Lombardo and Eichinger, 2000). Although primarily studied in leadership contexts (De Meuse et al., 2010), its core elements—learning from successes and failures, adapting to new conditions, and applying knowledge effectively—are highly relevant to higher education (Bedford, 2011; Jian, 2022; Murphy, 2021). This study examines how students reflect on performance, adjust strategies, and tackle unfamiliar tasks, drawing on established frameworks (Bedford, 2011). Howard (2017) highlights its key components: inherent potential, motivation, and adaptability.

In academic settings, learning agility enables students to thrive in dynamic environments, engage in self-directed learning, and address complex challenges like new technologies or interdisciplinary coursework (Bedford, 2011; De Meuse et al., 2010; Howard, 2017; Jian, 2022; Kim et al., 2018; Murphy, 2021). It encompasses cognitive flexibility, constructive social interactions, emotional intelligence, and reflective practice, all essential for meeting the demands of modern higher education (DeRue et al., 2012; Howard, 2017; Murphy, 2021). Lombardo and Eichinger (2004) outlined four dimensions of learning agility critical for student adaptability: mental agility, people agility, change agility, and results agility. Mental agility involves critical thinking and tackling complex academic problems, such as theoretical concepts or ambiguous tasks, from multiple angles. People agility reflects the ability to learn through collaboration, as seen in team-based coursework or peer study groups. Change agility entails experimenting with new methods, like innovative study techniques or unfamiliar teaching approaches. Results agility enables effective performance in challenging situations, such as demanding exams or sudden shifts in academic expectations. These dimensions support adaptability, fostering academic success and resilience in dynamic learning contexts (DeRue et al., 2012; Sopa and Pomohaci, 2016). Agile learners excel at applying lessons from experience to new challenges, analyzing problems, and synthesizing information while remaining inquisitive and reflective (De Meuse et al., 2010; Howard, 2017).

Learning agility is increasingly vital in higher education, enabling students to navigate complex and evolving academic environments. Research indicates that students with higher learning agility adapt effectively to digital learning platforms and changing pedagogical approaches, enhancing their academic engagement and success (Kim et al., 2018; Milani et al., 2021; Murphy, 2021). For example, students with strong digital competence and positive attitudes toward technology demonstrate greater learning agility, which supports achievement across diverse fields, such as music education, where constant adaptation to new techniques fosters persistence, self-efficacy, and academic success (Jian, 2022; Murphy, 2021).

Learning agility also promotes adaptability and resilience, allowing students to embrace change, learn from mistakes, and recover quickly from setbacks. This proved critical during academic transitions, such as the shift to online learning during the COVID-19 pandemic (DeRue et al., 2012; Mundiri et al., 2021; Novianti et al., 2023). Mundiri et al. (2021) found that agile students adjusted adeptly to virtual classrooms, maintaining academic performance despite disruptions. This adaptability not only improves academic outcomes but also supports emotional well-being by mitigating burnout during challenging academic periods (Novianti et al., 2023).

Although learning agility has been primarily studied in leadership contexts (De Meuse et al., 2010), its relevance to student development is gaining attention. Reed (2012) highlighted how military leaders use learning agility to navigate complex, high-pressure situations, a skillset transferable to academic settings where adaptability, critical thinking, and resilience are essential (De Meuse et al., 2010; Reed, 2012). Considered a stable trait across demographics, learning agility may be a stronger predictor of high performance than innate intellectual or personal attributes (Connolly, 2001; De Meuse et al., 2010). As higher education increasingly values creativity, critical thinking, and problem-solving, learning agility enables students to apply knowledge across academic and professional contexts, preparing them for lifelong learning and future challenges (De Meuse et al., 2010; Sopa and Pomohaci, 2016).

Despite its recognized importance, gaps persist in understanding learning agility in academic settings. DeRue et al. (2012) noted that its conceptual clarity remains underdeveloped, particularly when applied to student learning rather than organizational leadership. This study addresses this by examining learning agility through defined behavioral indicators in students (Bedford, 2011), but further research is needed to refine its definition and develop robust assessment tools. Longitudinal studies could explore how learning agility evolves over time and its long-term impact on academic and professional outcomes (Sopa and Pomohaci, 2016). Sopa and Pomohaci (2016) also suggested that early interventions to foster learning agility could produce more adaptive learners throughout their educational journey, offering insights into how educators can effectively nurture this trait.

### 2.2 Academic self-efficacy and student motivation

Academic self-efficacy, grounded in social cognitive theory, refers to a student’s belief in their ability to successfully perform academic tasks and achieve goals (Bandura, 1997). It plays a critical role in shaping student motivation, persistence, and achievement, as those with strong self-efficacy beliefs engage more effectively in learning strategies, exert greater effort, and persist through challenges (Honicke and Broadbent, 2016; Khine and Nielsen, 2022; Pintrich and De Groot, 1990; Schunk, 1991; Usher et al., 2019; Zimmerman, 2000). Self-efficacy influences how students approach academic tasks, encouraging enthusiasm over avoidance and promoting self-regulated learning strategies, such as goal setting, time management, and self-monitoring (Pintrich and De Groot, 1990; Schunk and DiBenedetto, 2014; Zimmerman, 2000). These strategies enable students to tackle difficult tasks, manage academic stress, and improve both academic performance and emotional well-being (Dogan, 2015; Schunk, 1991).

Research consistently links self-efficacy to academic success. It motivates students to set higher goals and use advanced learning strategies, leading to improved outcomes across educational contexts, including higher education, where it fosters positive learning-related emotions (Fokkens-Bruinsma et al., 2021; Putwain et al., 2013; Zimmerman, 2000). In medical education, for example, self-efficacy enhances self-regulation, contributing to better academic achievement (Wu et al., 2020; Zheng et al., 2021).

Additionally, academic self-efficacy significantly enhances students’ ability to cope with academic stress and challenges. Students with high self-efficacy often use adaptive coping strategies, such as effort regulation and goal setting, to manage pressures effectively, demonstrating resilience and grit (Crego et al., 2016; Komarraju and Nadler, 2013; Usher et al., 2019). Self-efficacy also supports motivation regulation, enabling students to handle the cognitive and emotional demands of learning, which is vital for success in higher education’s independent and demanding environment (Trautner and Schwinger, 2020). Social and environmental factors shape self-efficacy. Support from parents, peers, and teachers strengthens students’ confidence in their academic abilities, promoting success (Gebauer et al., 2020). In online education, perceived digital competence predicts self-efficacy and engagement, underscoring the importance of navigating digital platforms as education becomes more technology-driven (Kim et al., 2018; Neroni et al., 2022).

Despite its well-documented role in achievement, gaps remain in understanding self-efficacy. Longitudinal studies are needed to track how self-efficacy evolves and affects long-term academic paths (Schunk and DiBenedetto, 2014). Further research should explore its interactions with constructs like grit and future orientation to better understand its influence on success. Such insights are crucial for designing interventions to boost self-efficacy across diverse educational settings.

### 2.3 The role of academic buoyancy in student success

Academic buoyancy, understood as the ability to effectively manage routine academic setbacks such as disappointing grades, exam pressures, and other day-to-day academic stressors, is essential for maintaining student engagement and motivation (Martin and Marsh, 2008). It is crucial to distinguish this construct sharply from the broader concept of general resilience. General resilience typically refers to an individual’s capacity to overcome or adapt successfully to significant life adversities, such as trauma, major loss, or severe hardship (e.g., Herrman et al., 2011; Masten, 2001; Rutter, 1987). Whereas general resilience addresses larger, often life-altering challenges that can threaten fundamental adaptive systems, academic buoyancy specifically focuses on the capacity to navigate and bounce back from the frequent, comparatively smaller yet impactful obstacles that are inherent to the process of learning and assessment within the educational domain. These everyday academic hurdles are critical to sustaining motivation and well-being in the academic setting (Martin, 2013; Putwain et al., 2023; Kritikou and Giovazolias, 2022). Recognizing this distinction enables a clearer understanding of how students cope with the continuous demands of academic life (Collie et al., 2015) and informs the development of targeted interventions to support student success.

Frequent minor academic setbacks, such as failing an assignment or balancing multiple deadlines, can accumulate into chronic stress if not managed well (Martin and Marsh, 2020). Students with high academic buoyancy, however, are less likely to disengage, demonstrating resilience, persistence, and greater control over their academic outcomes (Bostwick et al., 2022; Collie et al., 2017; Comerford et al., 2015; Datu and Yang, 2021; Putwain et al., 2016). Buoyancy fosters adaptive emotional regulation and problem-focused coping, enabling students to maintain emotional balance and a positive outlook despite setbacks (Martin and Marsh, 2008; Putwain et al., 2022; Thomas and Allen, 2021). This approach not only supports immediate academic engagement but also enhances broader psychological resilience by minimizing negative emotional impacts on performance.

Academic buoyancy supports motivation, working alongside self-determined motivation and grit. Unlike grit, which drives long-term goal pursuit, buoyancy aids recovery from immediate academic setbacks, keeping students engaged and motivated in the short term (Aydın and Michou, 2020; Fong and Kim, 2021). This distinction highlights the value of buoyancy in addressing ongoing academic pressures. Buoyancy strongly predicts success across educational contexts. For example, Weißenfels et al. (2023) found a positive link between buoyancy and mathematics performance in secondary students, indicating better anxiety management in challenging subjects. Similarly, Lei et al. (2022) showed that buoyancy moderates the effect of self-efficacy on performance, helping confident, buoyant students sustain achievement under stress.

Beyond academic outcomes, buoyancy safeguards emotional well-being. Longitudinal research indicates a reciprocal link between buoyancy and achievement, where academic success strengthens buoyancy, creating a cycle that enhances resilience and performance over time (Collie et al., 2015). Students with high buoyancy manage negative emotions, such as frustration and anxiety, more effectively, preventing these from hindering academic progress (Putwain et al., 2022). Social support from family, peers, and teachers further boosts buoyancy, contributing to positive academic outcomes (Collie et al., 2017). Supportive academic environments are thus critical, providing resources for students to navigate daily stressors.

Despite substantial research, further exploration is needed. Martin and Marsh (2020) advocate for longitudinal studies to track buoyancy’s development and its long-term effects on academic success and emotional health. Investigating cultural and socioeconomic factors could also guide inclusive interventions to support diverse student populations.

### 2.4 The role of psychological well-being in education

Psychological well-being, as defined by Ryff (1995), includes autonomy, environmental mastery, personal growth, positive relations, purpose in life, and self-acceptance (Ryff, 2013; Ryff and Keyes, 1995; Ryff and Singer, 2008). This eudaimonic approach, emphasizing self-actualization over hedonic pleasure, is vital in education, where resilience, emotional regulation, and self-efficacy support student success (Amholt et al., 2020; Rüppel et al., 2015; Tang and Zhu, 2024). Research indicates that well-being enhances personal development and academic outcomes by fostering resilience and boosting cognitive and emotional engagement (Pietarinen et al., 2014; Van Ryzin et al., 2009).

Emotional and cognitive engagement are central to well-being, motivation, and academic performance. Enjoyment in learning promotes deeper understanding and well-being, while disengagement can cause frustration and anxiety, harming academic success and mental health (Martin et al., 2014; Pietarinen et al., 2014). Well-being strengthens intrinsic motivation, encouraging sustained effort toward academic goals and creating a positive cycle of engagement and achievement (Jiang and Tanaka, 2022; Martin et al., 2014).

Psychological well-being supports adaptive coping, protecting students from anxiety, depression, and burnout under academic stress through effective emotional regulation (Burris et al., 2009; Houben et al., 2015; Morales-Rodríguez et al., 2020). This resilience is crucial in high-pressure academic settings, reducing stress and promoting persistence (Fang et al., 2022). Autonomy support from staff enhances well-being and engagement, empowering students to manage challenges with motivation (Jiang and Tanaka, 2022). Social support also boosts academic buoyancy, aiding students in handling routine stressors and fostering long-term academic and emotional success (Af Ursin et al., 2021). These findings highlight the role of institutional support in strengthening psychological health and achievement.

Self-efficacy, the belief in one’s ability to meet academic demands, is central to well-being and resilience (Bandura, 1997; Fan and Cui, 2024). Bolstered by teacher relationships and grit, self-efficacy, alongside mindfulness and self-regulation, promotes emotional stability and stress management, enabling confident navigation of academic challenges (Fan and Cui, 2024; Tang and Zhu, 2024). Research emphasizes well-being’s impact on engagement, resilience, and motivation, but longitudinal studies are needed to examine its development and long-term effects on academic paths (Weiss et al., 2016). Exploring cultural and socioeconomic factors could inform tailored interventions for diverse students (Weiss et al., 2016). In conclusion, psychological well-being is essential for resilience, engagement, and academic success. Integrating well-being initiatives into curricula can enhance students’ emotional health and academic growth.

### 2.5 Interactions among learning agility, self-efficacy, buoyancy, and well-being

Learning agility, academic self-efficacy, academic buoyancy, and psychological well-being interact to shape academic performance and mental health. Each construct supports students in addressing challenges, maintaining motivation, and enhancing well-being. Recent studies explore their combined effects, focusing on the link between learning agility and self-efficacy, the mediating role of buoyancy, and their contributions to well-being.

Learning agility, the ability to learn from experience and adapt, strengthens academic self-efficacy, or confidence in achieving academic success (Bandura, 1997; Bedford, 2011). Students who adapt to challenges gain greater self-efficacy, while confident students persist in tasks, further enhancing agility in a reciprocal cycle (Jian, 2022; Lesmana and Ahmad, 2021; Yim and Lee, 2021).

Academic buoyancy, the capacity to recover from routine academic setbacks, mediates the relationship between learning agility, self-efficacy, and well-being. Unlike resilience, which targets major adversities, buoyancy addresses daily academic stress, sustaining engagement and motivation (Martin and Marsh, 2008). Students with high buoyancy manage stress effectively, improving performance and mental health, with social support amplifying the benefits of buoyancy (Af Ursin et al., 2021; Collie et al., 2015; Martin and Marsh, 2020; Morales-Rodríguez et al., 2020). This mediation proves vital in high-stress academic settings, where buoyancy reduces distress and fosters resilience (Tan et al., 2024).

Together, learning agility, self-efficacy, and buoyancy promote psychological well-being, which includes autonomy, environmental mastery, personal growth, positive relations, and self-acceptance (Ryff, 1995). Students with strong agility and self-efficacy report higher well-being, navigating challenges confidently and showing personal growth (Murphy, 2021; Ryff, 2013). Buoyancy further supports well-being by managing stress and preventing burnout and anxiety under academic pressure (Burris et al., 2009; Satıcı et al., 2024).

### 2.6 The purpose of the study

The purpose of this mixed-methods study is to explore the intricate relationships among learning agility, academic self-efficacy, academic buoyancy, and psychological well-being in the context of higher education. Specifically, the study seeks to examine how learning agility and academic self-efficacy predict students’ psychological well-being, with academic buoyancy acting as a mediating variable. Although these constructs have been studied individually in previous research (DeRue et al., 2012; Martin and Marsh, 2008; Ryff and Keyes, 1995), few studies have investigated how they interact within a unified framework to influence both academic success and psychological outcomes, particularly in non-Western educational contexts (Kim et al., 2018; Novianti et al., 2023).

This study also seeks to address gaps in the literature by employing a sequential explanatory mixed-methods design, integrating quantitative data from a large sample of undergraduate students with qualitative insights from in-depth interviews. The quantitative phase focuses on examining the predictive relationships between the study variables using Structural Equation Modeling (SEM), while the qualitative phase aims to explore students’ lived experiences, shedding light on how these constructs manifest in real academic scenarios. By combining both methodological approaches, the study provides a more comprehensive understanding of how learning agility and self-efficacy contribute to students’ resilience and mental health.

This research is particularly relevant in light of the increasing pressures faced by university students worldwide, especially in fast-paced, competitive academic environments like those found in China. By examining these relationships, the study contributes to the growing body of knowledge on the factors that promote academic buoyancy and psychological well-being, offering practical implications for educators and policymakers aiming to support student success and emotional health. Based on the literature reviewed, the following hypotheses are proposed:

1. *H1*: Learning agility will have a positive direct effect on academic buoyancy and psychological well-being.
2. *H2*: Academic self-efficacy will have a positive direct effect on academic buoyancy and psychological well-being.
3. *H3*: Academic buoyancy will positively predict psychological well-being.
4. *H4*: Academic buoyancy will mediate the relationships between (a) learning agility and psychological well-being, and (b) academic self-efficacy and psychological well-being.

## 3 Methods

### 3.1 Research design

This study adopted an explanatory sequential mixed-methods design, as described by Creswell and Plano Clark (2018). This approach involved collecting quantitative data first, followed by qualitative data to explain the quantitative results more comprehensively. The quantitative component aimed to investigate the relationships between learning agility, academic self-efficacy, academic buoyancy, and psychological well-being, utilizing SEM. The subsequent qualitative phase comprised semi-structured interviews to explore participants’ perspectives on how these constructs function in their academic experiences. The mixed-methods design allowed for both breadth and depth, with the quantitative results providing generalizable findings and the qualitative component offering rich, detailed insights (Creswell and Plano Clark, 2018).

### 3.2 Sample and data collection

The study included 804 undergraduate students (410 females, 394 males), aged 18 to 24, from two public universities in China. Participants were enrolled across various disciplines, including humanities, social sciences, engineering, and natural sciences. Recruitment involved university-wide announcements, emails, and class presentations. Eligibility criteria required (1) full-time enrollment in undergraduate studies and (2) willingness to participate in both the survey and, if selected, the interview. Students with diagnosed psychological disorders were excluded to control for confounding variables affecting well-being. A power analysis using G*Power 3.1 determined that a sample size of around 800 was sufficient for the study’s SEM analysis, ensuring a minimum power of 0.80 to detect medium effect sizes (Cohen, 1988).

Data collection proceeded in two phases. In Phase 1, participants completed an online survey assessing learning agility, academic self-efficacy, academic buoyancy, and psychological well-being. The survey, administered through an institutional platform, took about 25 min to complete, and participants received extra course credit to encourage participation. In Phase 2, 30 participants with varying levels of buoyancy and well-being were invited for semi-structured interviews. These interviews, conducted over a four-week period, were scheduled based on participants’ availability.

This study received ethical approval from the lead author’s institution’s University Ethics Committee. Participation was voluntary, and participants were informed of their right to withdraw at any time. All data were anonymized, and confidentiality was upheld throughout the study. Informed consent was collected prior to data collection to ensure ethical compliance.

### 3.3 Research tools

#### 3.3.1 Learning agility

Participants’ learning agility was assessed using a six-item scale adapted from Bedford (2011), measuring students’ ability to learn from experiences, reflect on errors, and adapt to new academic challenges on a five-point Likert scale from 1 (strongly disagree) to 5 (strongly agree). Sample items include, “I learn from mistakes quickly” and “I easily adapt to new academic challenges.” In this study, Cronbach’s alpha was 0.85, with CFA fit indices indicating good construct validity: *χ*^2^/df = 2.16, CFI = 0.95, TLI = 0.94, RMSEA = 0.05 [90% CI (0.03, 0.07)], SRMR = 0.04.

#### 3.3.2 Academic self-efficacy

The Academic Self-Efficacy Scale (ASES), developed by Pintrich and De Groot (1990), includes 22 items rated on a five-point Likert scale from 1 (strongly disagree) to 5 (strongly agree) to measure participants’ confidence in their academic abilities. Items such as “I believe I can succeed in difficult courses” and “I am confident in my ability to understand the most complex material” assess self-perceived competence. The scale was translated into Chinese and back-translated by two bilingual experts, with a pilot test confirming clarity and reliability (*α* = 0.83). CFA results were as follows: *χ*^2^/df = 2.34, CFI = 0.93, TLI = 0.92, RMSEA = 0.06 [90% CI (0.04, 0.08)], SRMR = 0.05.

#### 3.3.3 Academic buoyancy

Academic buoyancy was measured using a four-item scale from Martin and Marsh (2008), which assesses students’ ability to handle routine academic setbacks, such as low grades or exam stress, on a seven-point Likert scale from 1 (strongly disagree) to 7 (strongly agree). An example item is, “I do not let the stress of studying maths affect me negatively.” The scale’s internal consistency was 0.91, and CFA fit indices indicated acceptable model fit: *χ*^2^/df = 1.98, CFI = 0.94, TLI = 0.93, RMSEA = 0.05 [90% CI (0.03, 0.07)], SRMR = 0.04.

#### 3.3.4 Psychological well-being

The Scale of Psychological Well-Being (SPWB), developed by Ryff and Keyes (1995), measured psychological health across six dimensions: autonomy, environmental mastery, personal growth, positive relations, purpose in life, and self-acceptance, using 18 items rated on a six-point Likert scale from 1 (strongly disagree) to 6 (strongly agree). Example items include “I have a clear sense of purpose in life” and “I feel good about my personal growth.” The scale’s internal consistency was high (*α* = 0.88), with CFA fit indices as follows: *χ*^2^/df = 2.42, CFI = 0.92, TLI = 0.91, RMSEA = 0.06 [90% CI (0.04, 0.08)], SRMR = 0.05.

#### 3.3.5 Semi-structured interviews

In the qualitative phase, semi-structured interviews were conducted with 30 participants to gain a deeper understanding of their experiences with learning agility, academic self-efficacy, academic buoyancy, and psychological well-being. The interview protocol included open-ended questions such as “Can you describe a situation where your ability to adapt helped you overcome academic challenges?” and “How do you typically recover from academic setbacks?” Interviews lasted between 45 and 60 min and were conducted either face-to-face or via video conferencing. All interviews were audio-recorded with participants’ consent and later transcribed verbatim. Thematic saturation was reached after 27 interviews, but the final three were conducted to ensure completeness.

### 3.4 Data analysis methods

The quantitative data were analyzed using SEM, which was performed using AMOS version 27. Prior to analysis, the data were screened for outliers, missing values, and normality. Descriptive statistics, including means, standard deviations, and correlations, were computed to provide an overview of the relationships between variables. The mediation model was tested using a bootstrapping procedure with 5,000 resamples to estimate the indirect effects of academic buoyancy on the relationship between learning agility, academic self-efficacy, and psychological well-being. Model fit was evaluated using multiple fit indices, including the Comparative Fit Index (CFI), the Root Mean Square Error of Approximation (RMSEA), and the Standardized Root Mean Square Residual (SRMR). Based on established guidelines, CFI values above 0.90, RMSEA values below 0.08, and SRMR values below 0.08 were considered indicators of good fit (Hu and Bentler, 1999).

Thematic analysis was employed to analyze the interview data, following the six-phase approach outlined by Braun and Clarke (2006). Initially, transcripts were read repeatedly to ensure familiarity with the data, and open codes were generated based on patterns related to academic buoyancy and psychological well-being. Codes were then grouped into broader themes, such as “adaptation strategies” and “resilience in academic stress.” To ensure the trustworthiness of the qualitative data, two independent coders analyzed the interview transcripts. The initial coding process yielded an inter-rater reliability (Cohen’s kappa) of 0.84, indicating a high level of agreement between coders. Discrepancies were discussed and resolved through collaborative meetings, ensuring that the final themes accurately represented the participants’ experiences.

## 4 Results

### 4.1 Descriptive data and variable interrelations

Table 1 summarizes descriptive statistics for learning agility, academic self-efficacy, academic buoyancy, and psychological well-being. Participants reported moderate to high levels across all variables. Learning agility had a mean of *M* = 3.72 (SD = 0.67), indicating moderate adaptability to academic challenges. Academic self-efficacy was higher, with *M* = 4.05 (SD = 0.54), reflecting strong confidence in academic abilities. Academic buoyancy, measuring resilience to routine academic stress, showed *M* = 5.35 (SD = 1.12), suggesting effective stress management. Psychological well-being had *M* = 4.45 (SD = 0.82), indicating robust well-being. All measures demonstrated high reliability, with Cronbach’s alpha ranging from *α* = 0.83 (self-efficacy) to *α* = 0.91 (buoyancy) (Table 1).

**Table 1:** Descriptive statistics and reliability values.

Variable	Mean (*M*)	Standard deviation (SD)	Cronbach’s Alpha (*α*)
Learning agility	3.72	0.67	0.85
Academic self-efficacy	4.05	0.54	0.83
Academic Buoyancy	5.35	1.12	0.91
Psychological well-being	4.45	0.82	0.88

Pearson’s correlation analyses (Table 2) revealed significant positive relationships among all study variables, supporting the hypothesis that higher learning agility, academic self-efficacy, and academic buoyancy are positively associated with psychological well-being. Specifically, learning agility was significantly correlated with academic self-efficacy (*r* = 0.48, *p* < 0.001), academic buoyancy (*r* = 0.43, *p* < 0.001), and psychological well-being (*r* = 0.35, *p* < 0.001). Academic self-efficacy showed similarly strong correlations with academic buoyancy (*r* = 0.51, *p* < 0.001) and psychological well-being (*r* = 0.47, *p* < 0.001). Notably, academic buoyancy had the strongest correlation with psychological well-being (*r* = 0.60, *p* < 0.001), highlighting the central role of resilience in mental health outcomes.

**Table 2:** Pearson’s correlations.

Variable	1	2	3	4
1. Learning agility	–	0.48**	0.43**	0.35**
2. Academic self-efficacy	0.48**	–	0.51**	0.47**
3. Academic Buoyancy	0.43**	0.51**	–	0.60**
4. Psychological well-being	0.35**	0.47**	0.60**	–

^**^p < 0.001 for all correlations.

### 4.2 SEM analysis of hypothesized relationships

To assess the hypothesized relationships among the variables, SEM was conducted using AMOS version 27. The mediation model specified learning agility and academic self-efficacy as predictors, academic buoyancy as the mediator, and psychological well-being as the outcome variable. Maximum likelihood estimation was employed, and assumptions for SEM (e.g., multivariate normality and sample size adequacy) were met.

The SEM analysis demonstrated good model fit, with all key fit indices meeting acceptable criteria: *χ*^2^(142) = 289.75, *p* < 0.001, CFI = 0.951, TLI = 0.937, RMSEA = 0.056 [90% CI (0.047, 0.065)], and SRMR = 0.037. These indices indicate that the proposed mediation model fits the observed data well (Hu and Bentler, 1999). While the chi-square value was significant, this is common in larger samples, and the CFI, TLI, RMSEA, and SRMR values all fall within recommended ranges, suggesting the model adequately captures the relationships between the variables.

As seen in Table 3, the analysis revealed significant direct effects from both learning agility and academic self-efficacy to academic buoyancy. Learning agility positively predicted academic buoyancy (*β* = 0.423, SE = 0.05, *p* < 0.01), indicating that students who are more agile in their learning are more resilient in managing academic stressors. Similarly, academic self-efficacy significantly predicted academic buoyancy (*β* = 0.514, SE = 0.04, *p* < 0.01), underscoring that students with higher confidence in their academic abilities exhibit greater resilience when facing challenges.

**Table 3:** The path coefficients of SEM model.

Path	Standardized Coefficient (β)	SE	*p*-value
Learning agility → academic buoyancy	0.423**	0.05	< 0.01
Academic self-efficacy → academic buoyancy	0.514**	0.04	< 0.01
Academic buoyancy → psychological well-being	0.594**	0.06	< 0.01
Learning agility → psychological well-being	0.204*	0.07	< 0.05
Academic self-efficacy → psychological well-being	0.263*	0.06	< 0.05

*p < 0.05, **p < 0.01.

Additionally, academic buoyancy significantly predicted psychological well-being (*β* = 0.594, SE = 0.06, *p* < 0.01), suggesting that students who exhibit higher resilience in handling academic stressors report greater psychological well-being. Both learning agility (*β* = 0.204, SE = 0.07, *p* < 0.05) and academic self-efficacy (*β* = 0.263, SE = 0.06, *p* < 0.05) also had significant direct effects on psychological well-being, even when controlling for academic buoyancy. These results indicate that adaptability and confidence in academic tasks independently contribute to mental health, alongside the buffering effect of resilience provided by academic buoyancy.

A bootstrapping procedure with 5,000 resamples was used to test the mediating role of academic buoyancy in the relationships between learning agility, academic self-efficacy, and psychological well-being. The results revealed that academic buoyancy partially mediated the effect of learning agility on psychological well-being. Specifically, the indirect effect of learning agility on psychological well-being via academic buoyancy was significant [*β* = 0.25, SE = 0.04, 95% CI (0.18, 0.33)], confirming partial mediation. Similarly, the indirect effect of academic self-efficacy on psychological well-being through academic buoyancy was also significant [*β* = 0.31, SE = 0.05, 95% CI (0.23, 0.39)], highlighting that students with higher self-efficacy experience greater psychological well-being through their ability to recover from academic setbacks.

The total effects, which encompass both direct and indirect pathways, were also significant. The total effect of learning agility on psychological well-being was *β* = 0.45, SE = 0.06, *p* < 0.001, while the total effect of academic self-efficacy on psychological well-being was *β* = 0.57, SE = 0.06, *p* < 0.001. These results (Table 4) underscore the significant impact of both learning agility and academic self-efficacy on psychological well-being, with academic buoyancy serving as a critical intermediary in these relationships.

**Table 4:** Indirect and total effects for pathways in the model.

Pathway	Indirect effect (β)	SE	95% CI	Total effect (*β*)	SE	*p*-value
Learning agility → psychological well-being via academic buoyancy	0.25	0.04	[0.18, 0.33]	0.45	0.06	< 0.001
Academic self-efficacy → psychological well-being via academic buoyancy	0.31	0.05	[0.23, 0.39]	0.57	0.06	< 0.001

**Figure 1:**
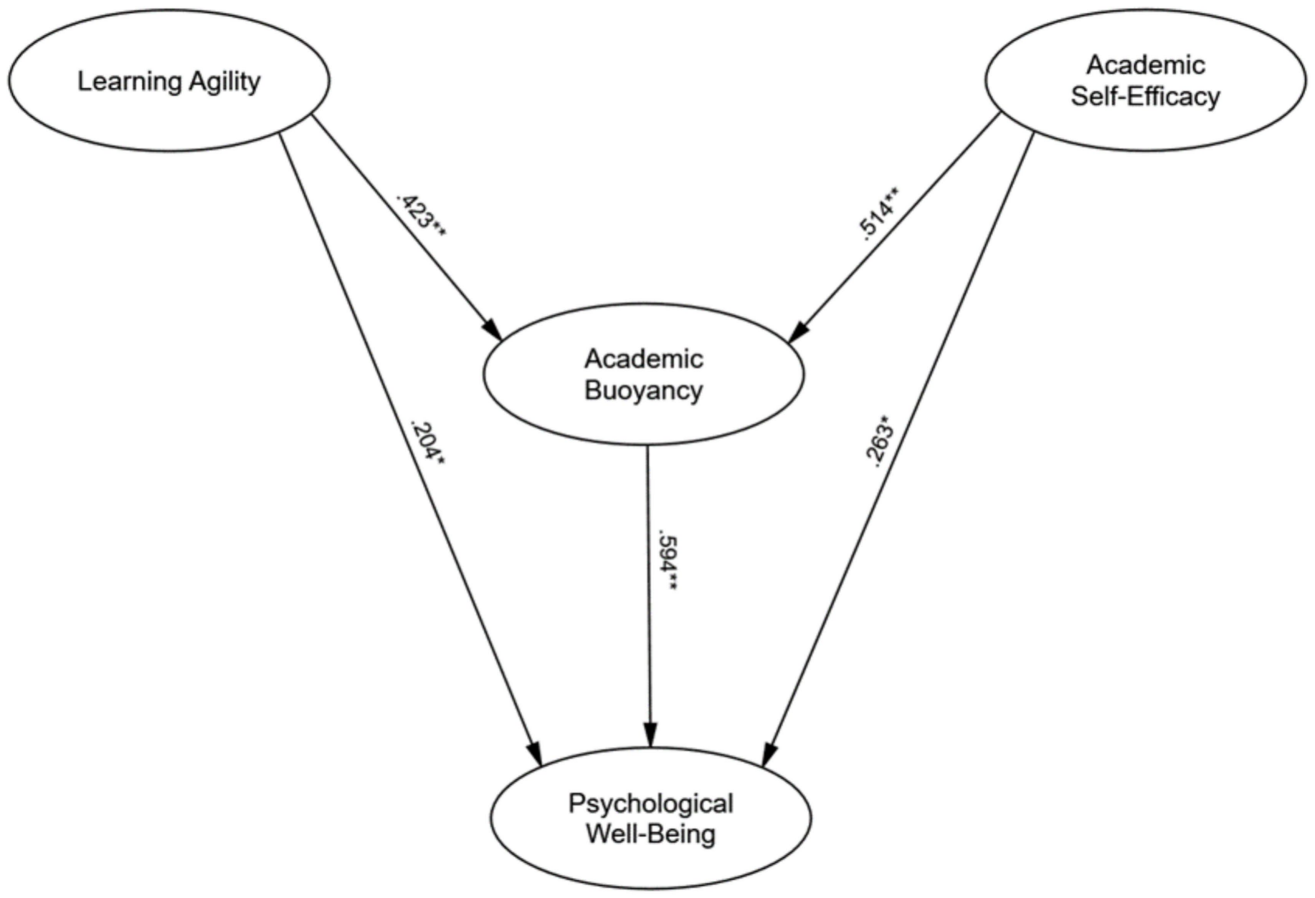
The model of psychological well-being.

These results demonstrate that both learning agility and academic self-efficacy are strong predictors of academic buoyancy and psychological well-being. Academic buoyancy serves as a significant mediator, linking learning agility and self-efficacy to psychological well-being, thereby reinforcing the importance of resilience in academic settings. Furthermore, both predictors have a direct impact on well-being, independently of academic buoyancy, underscoring the multifaceted ways in which adaptability and self-efficacy contribute to mental health in educational contexts (see Figure 1).

### 4.3 Gender comparisons in structural pathways

Given the balanced sample of male and female participants, a multi-group analysis was performed to assess whether the structural paths differed by gender. A model in which all path coefficients were constrained to be equal across genders was compared to a model in which the paths were freely estimated. The chi-square difference test was not significant (Δ*χ*^2^(5) = 3.21, *p* = 0.67), indicating no substantial gender differences in the relationships among learning agility, academic self-efficacy, academic buoyancy, and psychological well-being. Thus, the model operates similarly for male and female students, enhancing its generalizability.

Finally, to address potential common method bias, multiple procedural and statistical remedies were employed. First, the study utilized a time-lagged design where predictor variables (learning agility and academic self-efficacy) were collected at Time 1, while the mediator (academic buoyancy) and outcome variable (psychological well-being) were collected at Time 2. This temporal separation reduces the likelihood of common method variance influencing the relationships among the variables. Additionally, Harman’s single-factor test was conducted, revealing that a single factor accounted for only 28.4% of the variance, well below the 50% threshold, suggesting minimal risk of common method bias. Finally, a latent common method factor was included in the SEM analysis to account for any remaining variance due to common method bias, confirming that the bias did not significantly distort the findings.

### 4.4 Qualitative findings

The qualitative data were analyzed using Braun and Clarke’s (2006) six-phase thematic analysis approach. Through this analysis, the goal was to explore students’ personal experiences and perspectives concerning learning agility, academic self-efficacy, academic buoyancy, and psychological well-being. Thematic saturation was reached after 27 interviews, but all 30 were transcribed and analyzed to ensure data richness. Three key themes emerged: (1) Adaptive Learning Strategies, (2) Academic Resilience in the Face of Challenges, and (3) Psychological Well-Being as a Dynamic Process. Each theme is discussed below with a breakdown of subthemes, incorporating participant voices to illustrate the range of experiences.

#### 4.4.1 Theme 1: adaptive learning strategies

The theme of *Adaptive Learning Strategies* captures how students described their ability to adjust to new academic environments and challenges. Learning agility emerged as a key facilitator in academic success, particularly when students encountered unfamiliar or demanding tasks.

#### 4.4.2 Subtheme 1.1: reflecting on past mistakes

Many participants emphasized the value of reflecting on past mistakes as a means of improving future academic performance. For instance, one student shared how this reflective process helped her improve over time:

*“Whenever I get a low grade on an assignment, I sit down and figure out exactly where I went wrong. I’ve learned to embrace my mistakes and view them as opportunities to improve for next time.”* (Participant 14, female, 3rd year engineering).

This reflection, echoed by many participants, highlights learning agility in action. Students noted that their ability to critically assess their past performance allowed them to make necessary adjustments and adopt more effective strategies moving forward. As another student explained:

*“The first time I failed a midterm, I was devastated. But now, I take each setback as feedback. It’s part of the process, and I’ve gotten better at not letting it discourage me.”* (Participant 6, male, 2nd year sociology).

#### 4.4.3 Subtheme 1.2: seeking novel learning approaches

Participants also reported actively seeking out novel approaches to tackle difficult academic content, particularly when traditional methods failed. This adaptability was particularly evident in subjects outside their major disciplines. One participant remarked:

*“When I struggled with statistics, I did not just give up. I tried flashcards, group study sessions, even YouTube tutorials. I’m always experimenting to see what works best for me.”* (Participant 22, male, 2nd year economics).

This experimentation reflects the core of learning agility—an openness to new approaches. Several participants expressed the belief that this flexibility in learning methods was essential in adapting to unfamiliar challenges. Another student added:

*“I think it’s important to switch things up. If something does not work for me, I move on to something else. I do not stick with one way of learning.”* (Participant 10, female, 3rd year psychology).

#### 4.4.4 Theme 2: academic resilience in the face of challenges

The theme of *Academic Resilience* closely aligns with the concept of academic buoyancy, or students’ ability to “bounce back” from minor academic setbacks. Participants shared various strategies they used to manage stress and remain resilient in the face of everyday academic challenges.

#### 4.4.5 Subtheme 2.1: managing academic stress

Managing academic stress emerged as a common coping mechanism among participants. Many described learning how to better manage their time and seek support when needed. One student shared:

*“I used to get really stressed before exams, but now I plan my study schedule well in advance and make sure to take breaks. Having that balance helps me stay focused without burning out.”* (Participant 9, female, 4th year business administration).

Another participant echoed similar sentiments:

*“At first, I would just cram the night before an exam, but that did not work for me. Now, I spread out my studying and talk to classmates when I’m feeling overwhelmed.”* (Participant 18, male, 2nd year chemistry). These strategies underscore how students developed proactive approaches to mitigate academic stress, contributing to their academic buoyancy.

#### 4.4.6 Subtheme 2.2: viewing setbacks as temporary

Students’ ability to view academic setbacks as temporary rather than catastrophic was another significant aspect of their resilience. As one participant shared:

*“I did not do well in my final paper, but I reminded myself that it’s just one assignment. There’s always room for improvement, and I can bounce back.”* (Participant 5, male, 3rd year computer science).

This perspective allowed participants to maintain long-term focus on their academic goals, helping them to stay motivated despite short-term setbacks. Another student reflected:

*“A bad grade does not mean failure—it’s just a hiccup. I always remind myself that I can recover by putting in extra effort next time.”* (Participant 13, female, 1st year architecture).

#### 4.4.7 Theme 3: psychological well-being as a dynamic process

The theme *Psychological Well-Being* captures how students perceived their mental health as an evolving process, influenced by both academic pressures and personal factors.

#### 4.4.8 Subtheme 3.1: emotional regulation and well-being

Many participants discussed strategies they used to manage negative emotions, such as anxiety or frustration. One student shared how emotional regulation became a key factor in maintaining her well-being:

*“When I feel anxious, especially before presentations, I do breathing exercises and remind myself that it’s not the end of the world. Taking small steps to calm myself has made a huge difference.”* (Participant 17, female, 2nd year law).

Another student expressed how venting to friends helped him release academic pressure:

*“Sometimes all it takes is talking to a friend. It’s surprising how much better I feel after just getting things off my chest.”* (Participant 7, male, 3rd year computer engineering).

These reflections highlight the dynamic and personalized nature of emotional regulation strategies among students, emphasizing their impact on psychological well-being.

#### 4.4.9 Subtheme 3.2: purpose and self-acceptance

Many students tied their sense of purpose and self-acceptance to their overall psychological well-being. Several participants expressed that having a clear sense of purpose—academically or personally—provided them with the motivation to persevere through difficulties. One participant explained:

*“Knowing that I’m working toward a bigger goal, something I’m passionate about, gives me the drive to keep going, even when things get tough.”* (Participant 28, male, 4th year philosophy).

Self-acceptance also emerged as a key factor. As one participant stated:

*“I’ve learned to accept that I’m not perfect, and that’s okay. Once I stopped being so hard on myself, I found that I could enjoy my studies more and stress less.”* (Participant 21, female, 2nd year psychology).

#### 4.4.10 Additional insight: role of social support

An overarching theme that spanned across all three major themes was the critical role of social support. Students frequently cited support from friends, family, and professors as essential in navigating academic challenges and maintaining their psychological well-being. As one participant explained:

*“I would not have made it through last year without the help of my friends. We study together, encourage each other, and when things get tough, we are there to listen.”* (Participant 12, female, 3rd year biology).

This highlights the buffering effect of social support, reinforcing the importance of cultivating strong social networks to foster resilience and well-being in academic settings.

In summary, the qualitative analysis revealed that students perceive learning agility as a crucial factor in their academic success, particularly through reflection on mistakes and adaptive learning strategies. Academic buoyancy emerged as a key factor in helping students manage academic stress and setbacks, with students frequently employing both individual and social coping mechanisms. Finally, psychological well-being was found to be a dynamic, multi-faceted process, influenced by emotional regulation, a sense of purpose, and self-acceptance.

## 5 Discussion

This study set out to explore the interrelationships between learning agility, academic self-efficacy, academic buoyancy, and psychological well-being among undergraduate students. By employing a mixed-methods approach, we gained both breadth and depth in understanding how these constructs interact to shape students’ academic experiences and, centrally, their mental health outcomes. The findings from this study contribute to the growing body of literature on adaptive learning behaviors and resilience in academic contexts, offering practical insights for educators and institutions aiming to promote student well-being and create environments conducive to academic engagement.

### 5.1 Learning agility and academic self-efficacy

Our results indicate that learning agility positively influences academic self-efficacy, which is consistent with previous studies (Bedford, 2011; Jian, 2022; DeRue et al., 2012; Yim and Lee, 2021). Learning agility, defined as the ability to quickly learn from experiences and apply knowledge to new challenges (DeRue et al., 2012), appears to provide students with the confidence to approach academic tasks more effectively. The significant direct relationship between learning agility and academic self-efficacy in this study suggests that agile learners—those who adapt and experiment with new learning strategies—are more likely to feel confident in their academic abilities.

This finding aligns with Bandura’s (1997) social cognitive theory, which posits that self-efficacy develops through mastery experiences and the successful management of challenges. Agile learners engage in reflective practices, seeking feedback and adjusting their approaches to new tasks, which fosters a sense of competence and achievement (Lombardo and Eichinger, 2000; Schunk and DiBenedetto, 2014). Several participants in the qualitative phase of the study shared experiences of adapting to unfamiliar academic content and reflecting on past mistakes, which enhanced their confidence in tackling future challenges. For example, one student noted, “When I struggled with statistics, I tried various learning methods until I found what worked. Each time I improved, I felt more confident in my abilities” (Participant 22). This adaptability, a core component of learning agility, reinforces self-efficacy beliefs by enabling students to successfully navigate academic demands.

Moreover, learning agility appears to enhance academic engagement and persistence, particularly in dynamic academic environments (Kim et al., 2018; Murphy, 2021). The ability to quickly adjust to new academic settings—whether through adopting new learning technologies or adjusting to interdisciplinary coursework—likely plays a critical role in students’ confidence in their academic abilities. For instance, during the shift to online learning amid the COVID-19 pandemic, students who demonstrated learning agility adapted more effectively, as one participant stated, “I embraced the new online platforms quickly, which made me feel more capable and less stressed” (Participant 9). This relationship suggests that fostering learning agility can serve as a valuable strategy for enhancing self-efficacy among students, particularly in contexts where academic demands are continuously evolving (Mundiri et al., 2021; Novianti et al., 2023).

### 5.2 Academic buoyancy as a mediator

The findings also highlight the critical mediating role of academic buoyancy in the relationship between learning agility, academic self-efficacy, and psychological well-being. Academic buoyancy, which refers to the capacity to “bounce back” from everyday academic stressors (Martin and Marsh, 2008), emerged as a key mechanism linking adaptive learning behaviors to positive psychological outcomes. Both learning agility and academic self-efficacy were found to significantly predict academic buoyancy, which, in turn, contributed to enhanced psychological well-being. Notably, the mediation analysis revealed that academic buoyancy partially mediates these relationships, indicating that while learning agility and self-efficacy directly influence psychological well-being, a significant portion of their effect operates through enhancing students’ capacity to handle academic setbacks. This partial mediation suggests that academic buoyancy amplifies the positive impact of learning agility and self-efficacy on psychological well-being (Lei et al., 2022; Weißenfels et al., 2023).

This mediation effect supports previous research showing that academic buoyancy helps students manage routine academic stress, such as low grades and exam pressure, preventing negative emotional outcomes (Collie et al., 2015; Datu and Yang, 2021). In our study, students who demonstrated higher learning agility and academic self-efficacy were better equipped to handle these stressors, maintaining both motivation and resilience. This finding underscores the importance of developing students’ capacity for academic buoyancy, which not only helps them navigate academic challenges but also protects their mental health.

The qualitative data further illuminate this relationship. Participants frequently discussed how they viewed academic setbacks as temporary challenges rather than definitive failures, a key characteristic of academic buoyancy (Martin and Marsh, 2020). For instance, several students described how they developed strategies to manage academic stress, such as time management, seeking peer support, and maintaining a long-term perspective on their academic goals. One participant shared, “When I receive a low grade, I remind myself it’s just one assignment. I focus on what I can learn from it and how to improve next time” (Participant 5). These coping mechanisms, which align with the concept of academic buoyancy, helped students maintain engagement and motivation, even in the face of academic setbacks. This aligns with Thomas and Allen's (2021) findings that problem-focused coping strategies enhance students’ resilience and academic performance.

One student, for example, mentioned how they recovered from a poor exam performance by focusing on future opportunities to improve their overall grade. “I did not let one bad exam define me. Instead, I talked to my professor to understand my mistakes and made a plan to do better in the next one” (Participant 13). This ability to view setbacks as learning opportunities is integral to academic buoyancy and plays a protective role in sustaining psychological well-being.

### 5.3 Psychological well-being as an outcome

The results of this study also confirm the significant impact of learning agility, academic self-efficacy, and academic buoyancy on students’ psychological well-being. Psychological well-being, conceptualized through Ryff’s (1995) multidimensional model, includes critical dimensions such as autonomy, environmental mastery, personal growth, and self-acceptance. Our findings demonstrate that students who exhibit high levels of learning agility and self-efficacy experience greater psychological well-being, both directly and through the mediating effect of academic buoyancy.

These results align with existing literature that emphasizes the role of psychological well-being in promoting resilience, motivation, and academic engagement (Pietarinen et al., 2014; Van Ryzin et al., 2009; Tang and Zhu, 2024). In this study, academic buoyancy acted as a buffer against academic stress, contributing to students’ mental health by enabling them to recover from academic setbacks. As previous research has suggested, students who are better able to cope with minor academic stressors are less likely to experience burnout, anxiety, or depression (Burris et al., 2009; Morales-Rodríguez et al., 2020; Satıcı et al., 2024). The strong correlation between academic buoyancy and psychological well-being (*r* = 0.60) observed in this study underscores the critical role that resilience plays in maintaining positive mental health outcomes.

Additionally, the direct effects of learning agility and academic self-efficacy on psychological well-being—independent of academic buoyancy—suggest that these constructs contribute to students’ mental health through multiple pathways. Students who are agile learners and confident in their academic abilities are more likely to experience a sense of personal growth and environmental mastery, both of which are key dimensions of psychological well-being (Ryff and Keyes, 1995; Fan and Cui, 2024). These findings support Murphy’s (2021) assertion that learning agility promotes adaptability in dynamic academic environments, enabling students to manage academic demands while sustaining their well-being. Furthermore, academic self-efficacy appears to enhance students’ emotional resilience, helping them regulate negative emotions and maintain a positive outlook, even in the face of academic difficulties (Komarraju and Nadler, 2013; Schunk, 1991). The qualitative findings reinforce these results. Many participants described psychological well-being as a dynamic process influenced by their ability to adapt and cope with academic pressures. For example, one student noted, “Practicing mindfulness and accepting that I cannot control everything has helped me stay positive and focused” (Participant 17). This aligns with previous studies emphasizing the role of emotional regulation and self-acceptance in maintaining well-being (Houben et al., 2015; Shengyao et al., 2024).

## 6 Conclusion

This study confirms the interconnectedness of learning agility, academic self-efficacy, academic buoyancy, and psychological well-being in higher education. Crucially, academic buoyancy emerged as a key mediator, channeling the influence of adaptive learning and self-efficacy on student mental health. These findings underscore that by fostering these psychological resources, educators and institutions can promote student mental well-being, which in turn provides a strong foundation for academic engagement and potential achievement—competencies vital for navigating evolving educational landscapes and digital advancements.

The practical implications for educational practice are significant. Given the role of learning agility in enhancing academic buoyancy and well-being, higher education programs should integrate it as a foundational skill. Course design, moving beyond sole emphasis on content mastery, could cultivate cognitive flexibility and adaptive thinking through reflective exercises on performance and by using case-based learning or simulations that require agile responses to complex scenarios (Kim et al., 2018). Similarly, bolstering academic self-efficacy via instructional design and support initiatives is vital, given its direct contributions to buoyancy and well-being. Institutions could implement tailored programs such as mentorship with experienced peers or faculty and goal-setting workshops that break down complex tasks into manageable steps. Such programs can strengthen confidence in students, especially in demanding academic fields.

The emphasis of this study on the mediating role of academic buoyancy highlights that effectively managing everyday academic setbacks is critical for student engagement and mental health. Educational institutions should therefore embed practices that build this capacity into routine student development. Useful strategies include academic advising that normalizes setbacks as learning opportunities and the fostering of peer support networks where students share experiences and collectively navigate academic pressures. Finally, our findings advocate for institution-wide programs to reduce academic stress and promote psychological well-being. Since academic buoyancy is linked to managing daily stressors, integrating stress management workshops, mindfulness practices, and robust social support systems into student services is recommended. Cultivating collaborative learning environments can further enhance these efforts by fostering a sense of community that supports both academic and mental health outcomes, reinforcing the importance of social support for resilience.

## 7 Limitations and directions for future research

Despite its strengths, this study has several limitations that temper the interpretation and scope of our findings. First, our reliance exclusively on self-report measures for all constructs may introduce response biases, such as social desirability, or inaccuracies due to varying levels of self-perception among participants. While the instruments demonstrated high internal consistency, the subjective nature of self-reporting means that the magnitude of the relationships observed might be influenced by shared method variance or individual reporting styles. Future studies could enhance validity and mitigate these potential biases by incorporating objective measures or by employing multi-source data, for instance, instructor assessments of observable learning behaviors where feasible. Furthermore, while our study’s conceptual framework suggests pathways that may influence academic success, its primary empirical focus and outcome measurement centered on psychological well-being. Consequently, a notable limitation is the absence of comprehensive, objective measurement and in-depth analysis of academic achievement (e.g., grade point averages, standardized test scores, or course completion rates) within the current investigation. This restricts our ability to draw direct, empirically grounded conclusions from this dataset concerning the tangible impact of learning agility, self-efficacy, and academic buoyancy on students’ actual academic attainments. Future research should therefore prioritize the integration of such objective academic achievement data to more fully elucidate the practical implications of the proposed psychological pathways for both well-being and educational success.

Second, the characteristics of our sample necessitate caution regarding the generalizability and representativeness of the findings. The study focused on undergraduate students from two public universities in China. While this provides valuable insights into this specific educational context, it is important to acknowledge that cultural values, educational norms—such as the emphasis on perseverance and achievement—and support systems can differ significantly across other regions and countries. Consequently, the direct applicability of our findings to different cultural or educational settings may be limited. Future research should prioritize cross-cultural comparisons to evaluate the model’s robustness and adaptability across diverse student populations. Furthermore, the exclusion of students with diagnosed psychological disorders, a methodological step taken to control for pre-existing conditions that might confound the assessment of psychological well-being, means our sample does not fully represent the entire spectrum of the university student population. The dynamics of learning agility, self-efficacy, and academic buoyancy might operate differently for students already managing diagnosed mental health conditions. Therefore, future research specifically including and examining these student groups is essential for developing a more comprehensive understanding and ensuring support strategies are inclusive. Such broader research could also further illuminate how cultural factors influence the development and interplay of learning agility, self-efficacy, and buoyancy, thereby informing more culturally responsive interventions.

Third, the cross-sectional design of this study inherently restricts our ability to establish clear causal relationships between the variables or to track their developmental trajectories and reciprocal influences over time. Although our SEM analysis indicates that academic buoyancy mediates the relationship between learning agility, self-efficacy, and psychological well-being, the correlational nature of the data means we cannot definitively rule out alternative explanations or ascertain the direction of influence. A clear avenue for future inquiry thus involves employing longitudinal studies. Such an approach would be essential to track how learning agility, self-efficacy, and academic buoyancy (our specific measure of day-to-day academic resilience) develop and unfold throughout students’ academic journeys, providing more conclusive insights into their dynamic interplay and lasting impact on well-being and academic performance. Longitudinal designs would also allow researchers to identify key developmental periods when interventions to enhance these traits might be most beneficial.

Finally, a further limitation pertains to the study’s focused scope on specific academic and psychological constructs. While this focus allowed for an in-depth examination of the proposed pathways, we acknowledge that we did not comprehensively incorporate an analysis of a wider range of external factors that can significantly influence students’ psychological well-being. Variables such as students’ detailed socioeconomic status, the quality and nature of their family relationships, and their level of involvement in extracurricular activities were not explicitly measured or controlled for in our model. These socio-contextual elements undoubtedly play a role in shaping students’ overall life experiences, stress levels, and access to resources, which in turn can impact their psychological well-being within the higher education environment. For instance, a challenging family situation or financial pressures related to socioeconomic status might exert considerable stress on students, potentially moderating the effects of learning agility or academic buoyancy on their mental health. Conversely, strong social support from family or fulfilling extracurricular pursuits could serve as protective factors. Therefore, while our findings shed light on important intra-individual and academic pathways, the exclusion of these broader external variables means that our model does not capture the full ecological complexity of student mental health. Future research should endeavor to integrate these multifaceted external variables to develop a more holistic and nuanced understanding of the diverse factors that contribute to students’ psychological well-being and overall success in higher education.

## Data Availability

The data analyzed in this study is subject to the following licenses/restrictions: The data supporting the findings of this study are available upon reasonable request from the corresponding author, KH, at kou589406@sina.com. Due to confidentiality agreements, some restrictions may apply to the availability of qualitative interview transcripts. Requests to access these datasets should be directed to KH, kou589406@sina.com.
